# Treatment of Wastewater, Phenols and Dyes Using Novel Magnetic Torus Microreactors and Laccase Immobilized on Magnetite Nanoparticles

**DOI:** 10.3390/nano12101688

**Published:** 2022-05-15

**Authors:** Paula Andrea Peñaranda, Mabel Juliana Noguera, Sergio Leonardo Florez, Johana Husserl, Nancy Ornelas-Soto, Juan C. Cruz, Johann F. Osma

**Affiliations:** 1 Department of Electrical and Electronic Engineering, Universidad de los Andes, Cra. 1E No. 19a-40, Bogota 111711, Colombia; pa.penaranda1711@uniandes.edu.co (P.A.P.); mj.noguera10@uniandes.edu.co (M.J.N.); sl.florez10@uniandes.edu.co (S.L.F.); 2 Department of Civil and Environmental Engineering, Universidad de los Andes, Cra. 1E No. 19a-40, Bogota 111711, Colombia; jhusserl@uniandes.edu.co; 3 Laboratorio de Nanotecnología Ambiental, Escuela de Ingeniería y Ciencias, Tecnológico de Monterrey, Monterrey 64849, Mexico; ornel@tec.mx; 4 Department of Biomedical Engineering, Universidad de los Andes, Cra. 1E No. 19a-40, Bogota 111711, Colombia; jc.cruz@uniandes.edu.co

**Keywords:** dye, phenol, removal, bionanocomposites, laccase, magnetic nanoparticles, microreactor, torus

## Abstract

In this work, the design, manufacture, and testing of three different magnetic microreactors based on torus geometries (i.e., one-loop, two-horizontal-loop, and two-vertical-loop) is explored to increase the enzyme-based transformation of dyes by laccase bio-nanocomposites, improve the particle suspension, and promote the interaction of reagents. The laccase enzyme was covalently immobilized on amino-terminated silanized magnetite nanoparticles (laccase-magnetite). The optimal configuration for the torus microreactor and the applied magnetic field was evaluated in silico with the aid of the CFD and particle tracing modules of Comsol Multiphysics^®^. Eriochrome Black T (EBt) dye was tested as a biotransformation model at three different concentrations, i.e., 5 mg/L, 10 mg/L, and 20 mg/L. Phenol oxidation/removal was evaluated on artificial wastewater and real wastewater. The optimal catalytic performance of the bionanocomposite was achieved in the range of pH 4 to 4.5. A parabolic movement on the particles along the microchannels was induced by the magnetic field, which led to breaking the stability of the laminar flow and improving the mixing processes. Based on the simulation and experiments conducted with the three geometries, the two-vertical-loop microreactor demonstrated a better performance mainly due to larger dead zones and a longer residence time. Additionally, the overall dye removal efficiencies for this microreactor and the laccase-magnetite bionanocomposite were 98.05%, 93.87%, and 92.74% for the three evaluated concentrations. The maximum phenol oxidation with the laccase-magnetite treatment at low concentration for the artificial wastewater was 79.89%, while its phenol removal efficiency for a large volume of real wastewater was 17.86%. Treatments with real wastewater were carried out with a larger volume, equivalent to 200 biotransformation (oxidation) operating cycles of those carried out with dyes or phenol. Taken together, our results indicate that the novel microreactors introduced here have the potential to process wastewaters rich in contaminant dyes in continuous mode with efficiencies that are attractive for a potential large-scale operation. In this regard, future work will focus on finding the requirements for scaling-up the processes and evaluating the involved environmental impact indexes, economic performance, and different device geometries and processing schemes.

## 1. Introduction

Water pollution is one of the main environmental problems that mankind is facing over the coming few decades [1,2]. Industrial and agricultural processes are thought to be major causing agents due to the continuous discharge of large volumes of wastewaters to these aquatic environments [3]. Some of the most common discharged pollutants include pathogens, excess nutrients, suspended solids and sediments, pesticides, plastics, fertilizers, acids, detergents, pharmaceuticals, phenols, minerals, dyes and pigments, and heavy metals [4]. Phenolic compounds are mainly found in wastewater of many industries such as coal conversion, resin, plastic, and petroleum refineries. These compounds are toxic pollutants in industrial waste imposing risk to human health, and some of them are suspected carcinogens. Phenols and cresols are highly corrosive and toxic and can cause damage to the respiratory system, scarring of the skin, damage to gastrointestinal tracts, kidney failure, hematological changes, and nervous system depression [2,5]. The annual industrial production of synthetic or azo dyes approaches 70 million tons [6]. These xenobiotic chemicals are not normally encountered in nature and show high solubility in water and consequently, stand as one of the major sources of water pollution [7]. Due to their production ease, azo dyes are ubiquitously found around the world in many industries including textiles, leather goods, paper, plastics, foodstuffs, cosmetics, and candles [8]. Despite the industry’s efforts to couple wastewater treatment processes to their continuous production process, 90% of reactive textile dyes entering activated sludge sewage treatment plants will pass through unchanged [9]. As a result, between 30 to 150 thousand tons of dyes are discharged into water bodies, soil, and aquatic ecosystems annually [6].

The main methods used to treat water effluents containing azo compounds or phenolic compounds are coagulation/flocculation and precipitation, adsorption, flotation, membrane filtration, chlorine disinfection, bioflocculation, ion pair extraction, ultrasonic mineralization, electrolysis, ion exchange, advanced oxidation processes, sonication, photocatalysis, and ozonation [2,6,7]. Some of these processes, however, are difficult to operate, rely on costly feedstocks, require complex instrumentation and control schemes, exhibit limited versatility, and are negatively affected by other wastewater pollutants. Moreover, they can generate genotoxic or hazardous byproducts [10,11]. One attractive alternative to overcome some of these issues is the incorporation of enzyme-based biocatalysts as active components into treatment processes. This approach facilitates the degradation of the recalcitrant organic compounds, including the azo dyes and phenols, without introducing any extra toxic components, processing an ample range of pollutant concentrations, requiring shorter treatment times, and with low operation costs [12]. One of the most attractive families of enzymes for treatment of azo dyes are laccases [13]. These enzymes are oxidoreductases capable of oxidizing phenolic compounds into phenoxyl radicals, with the aid of four copper electrons in their structures [7,14].

Laccases have proven to catalyze the oxidation of a wide range of potential substrates, and they have high activity and specificity toward phenolic and non-phenolic aromatic compounds [15,16]. Nevertheless, free laccases are environmentally sensitive and difficult to recover from reaction media; have low long-term stability, costly isolation and purification processes, and limited large-scale applications due to their disposable use; and their catalytic activity shows a marked decrease in harsh environments [14,16]. Enzyme immobilization on different types of supports provides a technological route to compensate for many of these drawbacks, improve their stability, and allow their reuse in a cyclic reaction scheme [17,18].

Recently, laccases have been successfully immobilized on microscopic supports such as porous glasses [19,20], TiO_2_ [21], membranes [22], and microspheres [23]. Main immobilization methods include adsorption, self-immobilization, covalent binding, mesh embedding, microencapsulated embedding, and two-step combination. Yet, compared with conventional immobilization carriers, nanostructured materials are regarded as promising supports due to their small size, high surface area, and large surface-to-volume ratio [16]. MagnetiteM (Fe_3_O_4_) nanoparticles (MNPs) stand out as promising supports due to their large surface areas, superparamagnetism, ease of separation and recovery by applying external magnetic fields, and well-defined surface properties and morphology [24,25]. MNPs also stand out for their low toxicity, mature synthesis technology, and the possibility to be recycled without major changes in properties [16,19,26,27,28]. Thus far, MNPs have been exploited for the immobilization of several enzymes, including lipases, lactase, and glucosidase for degradation of pollutants [29].

Recent developments have focused on enabling incorporation of free and immobilized laccases into continuous treatment processes, which might be beneficial for low-cost industrial applications [13,30,31]. In this regard, continuous microreactor devices have been recently developed by taking advantage of important advances in the microfluidics field. One of the most attractive features of these devices is that they can carry out chemical processes with low reagent consumption due to the small sample volumes handled. There are different device configurations and peripherals that have been developed to assemble systems capable of complying with different analysis and functions including sampling, sample processing and in-line real-time monitoring, and processing of the collected data. With the advent of easier and cheaper ways of manufacturing at the microscale, the field of microfluidics has had an exponential growth and therefore has reached the sufficient maturity for its incursion into industrialization routes [32]. One of such tools is CFD simulations of the fluid flow and transport of objects within the microsystems [33]. With the simulation results, manufacturing takes shorter times and favor only prototypes with the highest performance, which can be further optimized with much less investment [34].

Here, we explore the design and manufacture of microreactors with toroidal topologies to enable the enzyme-based oxidation of phenols and dyes. We hypothesize that such microreactors are suitable for maximizing biotransformation processes due to the absence of dead volume, the efficient mixture of reagents, and the possibility of continuous reaction within the toroidal loop [35]. A first attempt to find an optimal configuration for the torus microreactor was explored in silico by analyzing mixing patterns and fluid dynamics. Low-cost device prototyping was conducted in polymethyl methacrylate using a laser cutting system and commercially available fittings for the assembly and subsequent testing. The microreactor’s potential for biotransformation of dyes was evaluated for laccases covalently immobilized magnetite (Fe_3_O_4_). The model reactions were the oxidation of the commercially available dye Eriochrome Black T (EBt) as well as phenol. To maintain the nanoparticles suspended during the treatment process and maximize contact between the components; one or two permanent magnets were coupled to the microreactor. Finally, the extent transformation of the azo molecules was examined in a real wastewater after measuring some water quality parameters and evaluating the removal of phenolic compounds from this water.

## 2. Materials and Methods

### 2.1. Materials

Polymethyl methacrylate (PMMA) sheets, also known as acrylic sheets, were purchased from Surtiacrylicos (Bogota, Colombia), Methyl methacrylate based-glue Veracril ^®^ was obtained from New Stetic (Guarne, Colombia), Ethanol (96%) was acquired from Expert Clean Colombia (Bogota, Colombia), and 345 mT Neodymium cylindrical magnets (ϕ: 6 mm × h 7 mm) were purchased from Constructor Mil Imanes (Bogota, Colombia). 2,2-azino-bis(3-ethylbenzothiazoline-6) sulphonic acid (ABTS), glutaraldehyde (25%), sodium hydroxide (NaOH) (98%), tetramethylammonium hydroxide (TMAH) (25%), and 3-aminopropyl-triethoxysilane (APTES) (98%) were purchased from Sigma-Aldrich (Burlington, MA, USA). Iron (II) chloride tetrahydrate (98%) (FeCl_2_*4H_2_O), iron (III) chloride hexahydrate (97%) (FeCl_3_*6H_2_O), phenol crystallized (99,5%) (C_6_H_6_O) (phenol) and dye Eriochrome Black T (EBt) (C.I. 14645) were obtained from PanReac AppliChem (Castellar del Valles, Spain).

### 2.2. Laccase

Laccases from *Pycnoporus Sanguineus* CS43 (EC 1.10.3.2) were obtained from tomato medium as described elsewhere [36]. In brief, mycelia were separated from the tomato medium supernatant after 10 days of culture by membrane filtration (0.2 μm RC Whatman). Then, it was concentrated by ultrafiltration with a tangential-flow filter (Membrane cut-off of 10 kDa, Sartorius Sartojet, Göttingen, Germany). The ultra-filtered sample was purified with a DEAE-cellulose ion exchange column eluted with 20 to 300 mM phosphate buffer pH 6.0 at a flow rate of 2 mL/min. The obtained laccase cocktail was collected and concentrated using an Amicon ultrafiltration cell (Membrane cut-off of 10 kDa, Merk Millipore, Burlington, MA, USA).

### 2.3. Synthesis of Magnetite Nanoparticles

Magnetite nanoparticles (Magnetite) were synthesized via coprecipitation by mixing 20 mL of 1M FeCl_2_ and 20 mL 2M FeCl_3_ under agitation at 1500 revolutions per minute (rpm) and 90 °C. Subsequently, 40 mL of 8M NaOH and 40 mL 2% (*v*/*v*) of TMAH were added to the mixture during 3.5 h at a flow rate of 12 mL/h. The obtained magnetite nanoparticles were purified by magnetic separation with the aid of a strong permanent magnet, then washed thoroughly with 2% (*v*/*v*) TMAH, and finally sonicated for 100 min using a VibraCell sonication system (Sonics, Newtown, CT, USA). The synthesized Magnetite exhibited an average hydrodynamic diameter of 88.59 nm with a polydispersity index of 0.182 as determined by Dynamic Light Scattering (DLS) analysis with the aid of a Zetasizer Nano ZS, (Malvern, UK) as previously reported by Lopez-Barbosa et al. (2020) [37]. Microscopic inspection of the Magnetite structure, morphology, and shape was achieved via transmission electron microscopy (TEM) in a Tecnai F30 instrument (FEI Company, Fremont, CA, USA)

### 2.4. Enzyme Immobilization

#### 2.4.1. Enzyme Immobilization on Magnetite Nanoparticles

A nanoparticle suspension (5 mg/mL) was buffered using a NaOH 1M solution until reaching pH 11, then sonicated for 10 min. Next, 50 µL of 2% (*v*/*v*) TMAH was pipetted, and the resulting reaction mixture sonicated for 10 min. Silanization was carried out by adding 50 µL of 2% (*v*/*v*) APTES, and then the mixture was sonicated again for 20 min. Subsequently, 50 µL of 2% (*v*/*v*) glutaraldehyde was added to the mixture as the crosslinker and left to react for 30 min. Finally, 50 µL of 960 U/L laccase enzyme was added and left overnight to immobilize on the surface of the MNP via covalent bonding. The resulting bionanocomposites (laccase-magnetite) were recovered with the aid of a strong permanent magnet and washed thoroughly with Milli Q water. Laccase immobilization on magnetite was confirmed by Fourier transform infrared spectroscopy (FT-IR) aided by an A250/D FTIR-ATR (Bruker, Bremen, Germany). The resulting laccase-magnetite exhibited an immobilization ratio of 0.0096 U/g of laccase activity per MNP mass, and 13 µg/g of laccase mass per MNP mass.

#### 2.4.2. Effect of pH and Temperature on Enzymatic Activity

Stability of free (free-laccase) and immobilized laccase (laccase-magnetite) bionanocomposites was examined in phosphate–citrate buffer solutions at pH values of 2.0, 3.0, 4.0, 4.5, 5.0, 6.0, 7.0, and 10.0, at 25 °C and at different temperatures ranging from 30 to 70 °C, at pH 4.0. Laccase activity, of free and immobilized laccase, was determined spectrophotometrically as described by Moilanen et al. [11] at 436 nm using a GENESYS 10S UV-Vis v4.004 2L5R078128 (Thermo Fisher Scientific, Waltham, MA, USA). One activity unit was defined as the amount of enzyme that oxidized 1 µmol of ABTS per min. The activities were expressed in U/L. All measurements were carried out in triplicate.

### 2.5. Microreactors Design and Manufacture

#### 2.5.1. Microreactor Geometry Design and Simulation

**Figure 1:**
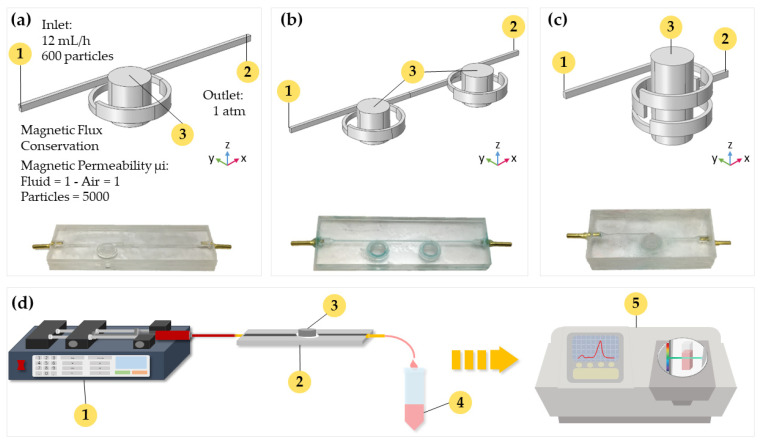
(**a**) Simulation geometry and actual picture of one-loop microreactor. (**b**) Simulation geometry and actual picture of the two-horizontal-microreactor. (**c**) Simulation geometry and actual picture of the two-vertical-loop microreactor. (**d**) Schematic of the experimental setup for the biotransformation process. The numbers 1, 2, 3, 4, and 5 in the figure correspond to the solution injection, microreactor, permanent magnet, treated sample, and absorbance analysis, respectively.

Three different geometries for the torus microreactor were studied in silico via Comsol Multiphysics 5.3^®^ (Stockholm, Sweden). Figure 1 shows such geometries, namely, one-loop, two-horizontal-loop, and two-vertical-loop microreactors. All microreactors were designed to work under continuous operation, with a wastewater continuous input flow through the inlet that travels through the microchannel until reaching the outlet. During its trajectory, wastewater meets with the laccase-magnetite bionanocomposites that are contained inside the microchannel by the effect of the magnetic field of a permanent magnet or magnets coupled to the systems, depending on the design. Wastewater is then treated by the laccase-magnetite and continue its trajectory to the outlet of the microreactor, while laccase-magnetite is retained inside the microreactor. To study the behavior of each microreactor design, the Computational Fluid Dynamics (CFD) and Magnetic Field no current (MF) modules of Comsol^®^ were coupled to simultaneously simulate the hydrodynamics and impact of the imposed magnetic fields, respectively. To evaluate the impact of magnetic fields on the transport of the individual nanoparticles, the Particle Tracing (PT) module was implemented solely for the one-loop microreactor.

The laminar flow module (Equation (1)) was used here to describe the fluid flow due to the low Reynolds number (*Re*) calculated for the microreactors (*Re* = 4.4). The inflow to the microreactor was 12 mL/h, while the output pressure was set to 1 atmosphere. Density and viscosity of water were assumed as the properties of the flowing fluid. The non-slip boundary condition was imposed at the walls of the microchannels. The imposed boundary conditions are summarized in Figure 1. MF was used to simulate the magnetic field generated by a neodymium permanent magnet (Equations (2) and (3)). An additional domain representing the surrounding air was added to the computational domain. Air and water were assumed with magnetic permeability of 1. These two physics were solved simultaneously in a stationary study with the direct solver PARDISO:

(1) $$0=-\nabla P+\mu \nabla^{2}V$$

(2) $$B=\mu_{0}\mu_{r}H$$

(3) $$B=\mu_{0}\mu_{r}H+B_{R}$$

In these equations, ∇*P* is the pressure gradient in the fluid, *µ* is the viscosity of the fluid, and *V* is the velocity of the fluid. For the equations of MF, *B* is a magnetic flux density, *μ*_0_ is the magnetic permeability of the vacuum, *μ_r_* is the magnetic permeability of the fluid, *H* is the magnetic field, and *B_R_* is the remanent flux density of the neodymium magnet. The PT module was used to study the dynamics of the magnetic particles within the microreactor. The particle diameter was assumed as 1.2 µm with a density of 5180 kg/m^3^. The particle size was decided according to our own previous experimentation that demonstrated the presence of agglomerations of NPs forming clusters (data not shown). Additionally, the magnetic permeability of the particle was set to 5000 H/m. The particles were delivered at the main inlet of the device. The PT was solved in a time-dependent study for 10 s using 0.1 s steps. In this case, a projected conjugate gradient iterative solver was chosen due to the high demand for computational resources. Additionally, a parametric analysis was performed to evaluate the impact of the remanent flux density of the neodymium magnet on the particles transport. Equations (4)–(7) describe the forces experienced by a particle. This study considered the interactions between the particles and the fluid (drag force) and the magnetic attraction between particles. Particle-particle interactions, lifting force, and Brownian motion were disregarded to avoid dealing with unnecessary complexity that provides little extra information at the expense of exceedingly large computational resources:

(4) $$F_{D}=\frac{1}{\tau_{P}}\;m_{P}\left(U-V\right)$$

(5) $$\tau_{P}=\frac{\rho_{P}d_{P}^{2}}{18\mu }$$

(6) $$F_{mp}=2\pi r_{p}^{3}\mu_{0}\mu_{r}k\nabla H^{2}$$

(7) $$k=\frac{\left(\mu_{rp}-\mu_{r}\right)}{\left(\mu_{rp}+2\mu_{r}\right)\;}\;$$

In these equations $F_{D}$ is the drag force, $m_{P}$ is the mass of the particle, *U* is the velocity of the particle, *V* is the fluid velocity, $\rho_{P}$ is the density of the particle, and $d_{p}$ is the particle diameter. *F_mp_* is the magnetophoretic force, $r_{P}$ is the particle radius, and $\mu_{rp}$ is the magnetic permeability of the particle. As described before, the meshing was subjected to a convergence analysis to determine the minimum number of elements necessary to arrive to a meaningful solution. For this study, five random measurement points in the microchannel were selected along the computational domain, and the change in magnitude of speed was evaluated as the number of mesh elements was increased. As a convergence criterion, it was determined that the velocity magnitude change obtained for a meshing level and the next one was below 3%. An unstructured mesh with 60,000 tetrahedral elements was therefore generated. A stationary study was then run with the direct solver PARDISO that allows to parallelize processes solving large symmetric or structurally symmetric dispersed linear systems of equations in shared memory multiprocessors [38].

#### 2.5.2. Manufacture of the Torus Microreactors

Device prototyping was conducted by engraving the microchannels (1 mm of depth) on PMMA slides of 2.5 mm thickness with an area of 75 mm × 25 mm. The proposed torus geometries were cut with the aid of a laser cutter system Speedy 100, 60 W (TROTEC, Germany). Proper assembly and sealing were achieved by gluing the slides with ethanol followed by heating at 105 °C for 8 min. The device was then maintained under constant pressure with the aid of a home-made press. Inlets and outlets were equipped with commercially available fittings to facilitate connection to pumping devices (e.g., syringe pumps) (See Figure 1).

### 2.6. Dye Decolorization Studies

#### 2.6.1. Experimental Tests for Decolorization of Dyes

EBt dye was used as the model compound to study dye treatment using MNP or laccase-magnetite inside the microreactors at pH 5.5 and three different dye concentrations, namely, 5 mg/L, 10 mg/L, and 20 mg/L. The setup consisted of a microreactor previously loaded with magnetite or laccase-magnetite, neodymium permanent magnets placed in the corresponding slots, and 5 mL of Ebt solution, at previously described concentrations, pumped through the inlet at a constant rate of 12 mL/h for 25 min. Prior to the decolorization experiments, 5 mg of MNP or laccase-magnetite (representing 4.85 × 10^−5^ U of laccase activity) was introduced to each microreactor in the presence of the neodymium permanent magnets, of 349.23 mT, to retain the bionanocomposites at the reaction loops. Samples were analyzed spectrophotometrically by measuring the absorbance peak at 545 nm, which is the maximum absorbance for EBt, and the absorbance area of the visible spectrum in the range between 400 and 700 nm (See Figure 1). All measurements were carried out in triplicate.

(8) $$\%\;Removal=\frac{Abs_{Dye\;Control}-Abs_{Sample}}{Abs_{Dye\;Control}}\;$$

The dye decolorization was calculated as removal percentage according to Equation (8), where the $Abs_{Dye\;Control}$ is the average absorbance (at the absorbance peak or the area under the absorbance curve, respectively) of the dye at each concentration prior to entering the microreactor, and the $Abs_{Sample}$ is the absorbance at the outlet of each replica.

#### 2.6.2. Treatment of Artificial Wastewater

Artificial wastewater (AW) (pH 4.42) containing three different concentrations of phenol, namely, 5 mg/L, 10 mg/L, and 20 mg/L was prepared. A total of 5 mg of laccase-magnetite (representing 4.85 × 10^−5^ U of laccase activity) was introduced to each microreactor in the presence of the neodymium permanent magnets to retain the bionanocomposites at the reaction loops. Each microreactor was then infused with 5 mL of each artificial wastewater solution at a constant rate of 12 mL/h for 25 min. Effluent samples were analyzed spectrophotometrically by measuring the absorbance peak at 270 nm, which is the maximum absorbance for phenol, and the absorbance area in the range between 190 and 1100 nm. All measurements were carried out in triplicate.

Phenol oxidation was calculated as a percentage of change in the phenol concentration, according to Equation (9). Where the $Abs_{AW\;Control}$ is the average absorbance (at the absorbance peak or area under the absorbance curve, respectively) of phenol at each concentration previous to entering the microreactor, and the $Abs_{Sample}$ is the absorbance of each replica, while $Abs_{Total\;conversion}$ is the absorbance of the total oxidized phenol. A value of zero in the percentage corresponds to a situation of no noticeable change in the phenol contents while negative values represent a reduction in such a concentration mainly due to physical phenomena (e.g., adsorption). Additionally, positive values might be explained by the polymerization of phenol upon oxidation by the Laccase molecules.

(9) $$\%\;Oxidized\;Phenol=\frac{Abs_{Sample}-Abs_{AW\;Control}}{Abs_{Total\;conversion}-Abs_{AW\;Control}}$$

#### 2.6.3. Treatment of Real Wastewater

Real Wastewater (RW) samples were collected from the laboratories of the School of Medicine at los Andes University (Bogota, Colombia). RW was then filtered through filter paper Munktell grade 3 HW with a pore diameter of 110 nm (Ahlstrom, Finland) to remove solid particles and some colloids that could interfere with the process. RW was then classified as untreated-RW (U-RW) and characterized. In contrast, treated-RW (T-RW) corresponds to U-RW after treatment through the laccase-magnetite system inside the two-vertical-loop microreactor. A total of 5 mg of laccase-magnetite (representing 4.85 × 10^−5^ U of laccase activity) was introduced to the microreactor prior to treatment, as explained previously. One liter of U-RW was pumped into the microreactor at a constant rate of 12 mL/h. T-RW was then collected at the outlet of the microreactor.

Samples of U-RW and T-RW were analyzed to quantify phenol, biochemical oxygen demand (BOD), NH_3_-nitrogen, and total Kjeldahl nitrogen concentrations. BOD was analyzed using the Standard Method 5210B; NH_3_-nitrogen was analyzed by Standard Method 4500-NH_3_-C; total nitrogen was evaluated by Standard Method 4500-N. Phenol was selected as a role contaminant, as it is susceptible to oxidation by the immobilized laccase molecules. Nitrogen was analyzed due the presence of amine groups in the chemical structure of immobilization linkers. Finally, the biochemical oxygen demand was quantified to estimate the amount of dissolved organic matter present in the samples, which can be partially oxidized by laccase enzymes.

The removal ratio for each of the parameters above was calculated according to Equation (10), where the $X_{U-RW}$ is the value obtained for each parameter in the untreated sample (U-RW), and $X_{T-RW\;}$ is the value of the same parameter after treatment (T-RW).

(10) $$Removal\;Ratio\;\left[\%\right]=\frac{\left(X_{U-RW\;}-X_{T-RW\;}\right)}{X_{U-RW\;}}\;$$

## 3. Results and Discussion

### 3.1. Characterization of Laccase Immbolized on Magnetite

**Figure 2:**
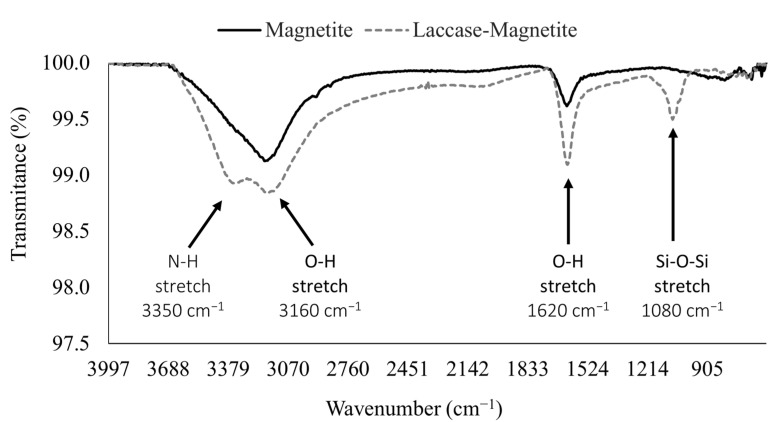
FTIR analysis of magnetite nanoparticles and laccase-magnetite nanobiocomposites, showing the identified peaks at 1080, 1620, 3160 and 3350 cm^−1^.

FTIR was carried out to confirm the immobilization of laccase on magnetite nanoparticles (laccase-magnetite) (Figure 2). Bare magnetite exhibited two bands at 1620 and 3160 cm^−1^ related to the vibration of the hydroxyl groups on the surface of the magnetite ν (Fe-OH) [39,40]. Laccase-magnetite nanobiocomposites presented a peak at 3350 cm^−1^ attributed to the NH stretching vibration of laccase amines and a peak at 1080 cm^−1^ characteristic of the Si-O-Si bond resulting after silanization [39].

**Figure 3:**
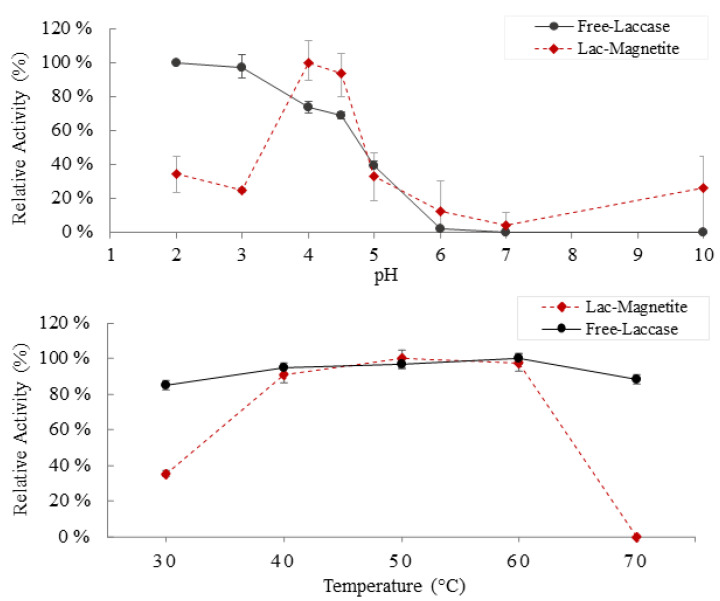
Effect of pH and temperature on enzyme activity: (●) free-laccase and (♦) laccase-magnetite.

We evaluated the impact of pH and temperature on enzyme activity (Figure 3). Optimal activity for the free-laccase was observed under acidic conditions (maximum activity at pH 2). Similar optimal values between pH 2–4 were reported previously [41]. In contrast, for pH values of 6 and above, the activity decreases and becomes almost negligible. In the case of the bionanocomposites, maximum activity was observed at pH 4, followed by a considerable decrease for pH values of 6 and above. The change in the optimal pH for Laccase-Magnetite to higher values is likely caused by an uneven concentration of the H^+^ and OH^−^ ions between the support matrix and the bulk solution [33]. Regarding the effects of temperature on the activity of the enzyme, both the immobilized laccase and the free laccase showed more than 85% of relative activity between 40 and 60 °C; in both cases (free-laccase and laccase-magnetite), there is a pronounced deactivation effect above 60 °C, being slightly higher for the immobilized laccase, losing around 15–30% of the relative activity.

### 3.2. Microreactor Geometry Design and Simulation

**Figure 4:**
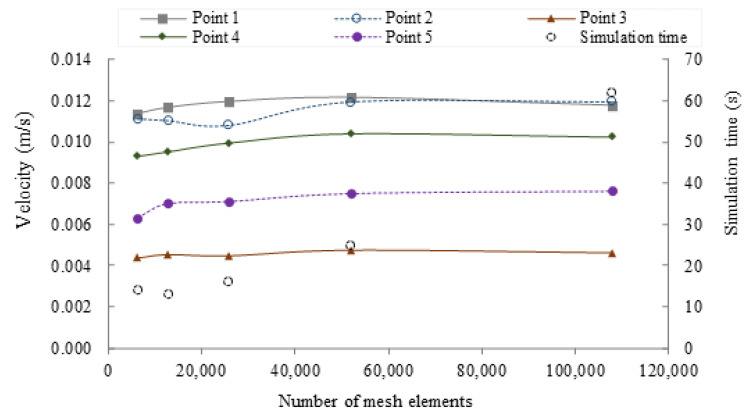
Mesh convergence analysis for the one-loop torus microreactor. Five points along the computational domain were selected for the analysis and plotted as a function of number of mesh elements. Additionally, the simulation time for the different levels of meshing is presented as dotted circles.

Figure 4 shows the convergence plot for a one-loop microreactor. All the simulations were carried out with a minimum of 60,000 mesh elements for the fluid domain to assure the system convergence.

**Figure 5:**
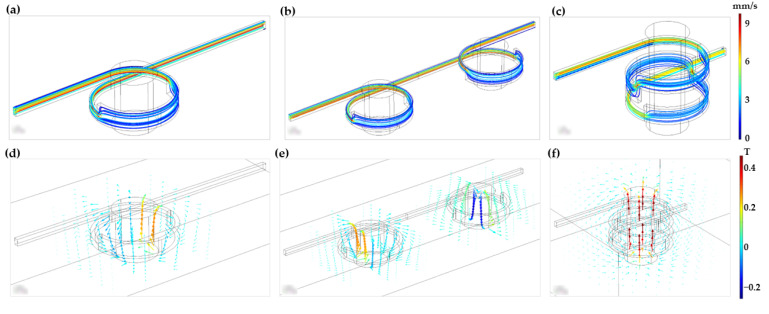
(**a**) Velocity profile of one-loop microreactor. (**b**) Velocity profile of two-horizontal-loop microreactor. (**c**) Velocity profile of two-vertical−loop microreactor. (**d**) Magnetic field flux of one-loop microreactor. (**e**) Magnetic field flux of two-horizontal-loop microreactor. (**f**) Magnetic field flux of two-vertical-loop microreactor.

The velocity profiles and magnetic field fluxes for the three proposed devices are shown in Figure 5. Dead zones of low velocities (blue color) are observed in the three configurations; however, the two-vertical-loop microreactor shows a larger dead zone compared to the other 2 devices. This can be explained by the relatively important changes in height as the fluid passes through the loops. In addition, the fluid decelerates because of changes in the cross-sectional area of the microchannels. This velocity reduction is beneficial for biotransformation purposes as the contact time of the bionanocomposites with the fluid is likely to be significantly exacerbated.

The magnetic field flux results show a uniform distribution around the magnet for the case of the single loop (Figure 5d). For the two magnets arranged in a horizontal configuration (Figure 5e), there is no interaction between the field lines of the magnets. Finally, the configuration of two-vertical-loop (Figure 5f) showed the greatest field interaction, and consequently superior chances to retain the bionanocomposites within the loops.

**Figure 6:**
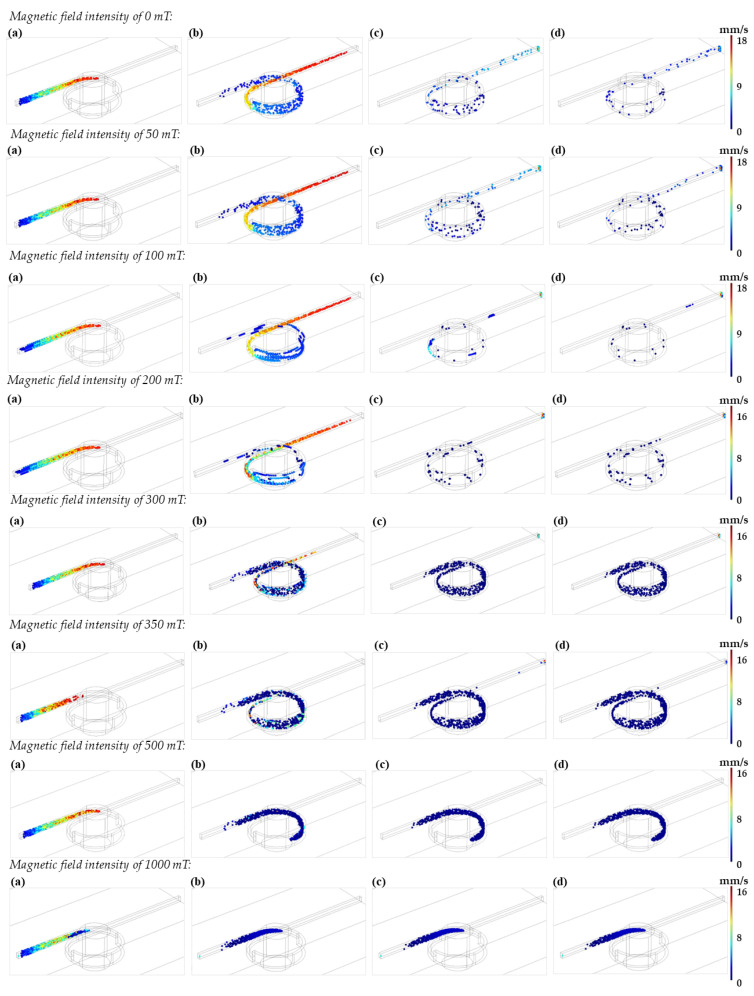
(**a**) Particle distribution in the one-loop microreactor after 1 s. (**b**) Particle distribution in the one-loop microreactor after 5 s. (**c**) Particle distribution in the one-loop microreactor after 15 s. (**d**) Particle distribution in the one-loop microreactor after 25 s.

The time evolution of the particle’s transport within the one-loop microreactor was studied under varying intensities of the applied magnetic field (i.e., 0, 50, 100, 200, 300, 350, 500, and 1000 mT). The simulations were conducted for a total time of 30 s with data collection at 1, 5, 15 and 25 s (See Figure 6). For these simulations, each particle trajectory is random and driven by the exerted drag and magnetophoretic forces. As the particles are attracted to the magnet and due to the interplay of involved forces in the loop, they follow a parabolic trajectory until finally sticking to the wall of the microreactor channel. For some particles, the magnetic field will not be enough to retain them within the loop. These unique trajectories have been exploited by others for the separation and manipulation of magnetic particles within microchannels [42]. In our case, the movement is enough to perturb the laminar flow, thereby generating mixing patterns that are useful to promote the intimate interaction of the nanoparticles with other components present in the solution. This was also evidenced by a better suspension of the nanoparticles as the magnetic field was increased.

Total retention of the particles was achieved for the fields of 500 and 1000 mT, as the particles remain statically attached to the walls of the microreactor. To support this result, the particle’s loss and retention ratio were analyzed by counting the particles leaving the system through the microreactor’s outlet during the 30 s of simulation (See Table 1). The magnetic field applied experimentally by the magnet was of 349.23 mT, which is close to the one simulated at 350 mT. In this case, the particle’s retention approached 96.83% while the loss ratio was 3.17%. The actual particle’s loss ratio obtained experimentally was between 13–20%, which was closer to the results obtained in silico at 300 mT where the retention ratio was 82.67%. In this case, the loss ratio approached 17.33%. The reduction in the actual strength of the magnetic field can be explained by the marked difference in the medium surrounding the fluid computationally (i.e., air) and the actual medium (i.e., PMMA walls), which attenuates the applied magnetic field.

### 3.3. Decolorization and Degradation Studies

#### 3.3.1. Experimental Tests for Decolorization of Dyes and Degradation of Phenols

**Figure 7:**
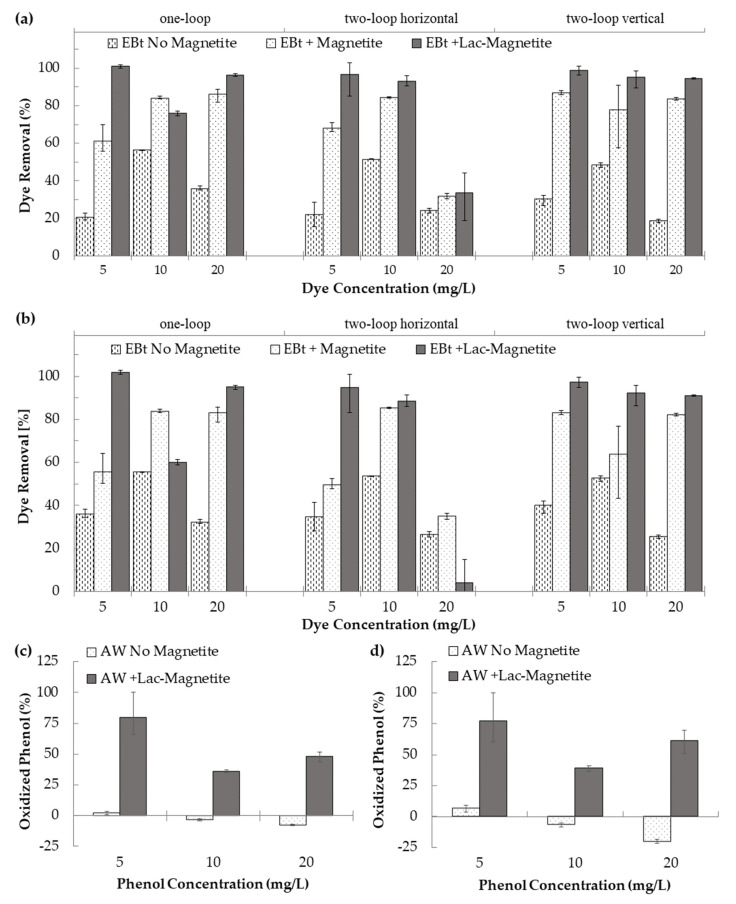
(**a**) Removal of Eriochrome Black T for the three microreactor configurations as estimated by absorbance peak. (**b**) Removal of Eriochrome Black T for the three microreactor configurations as estimated by absorbance area. (**c**) Percentage of oxidized phenol for the two-vertical-loop microreactor as estimated by absorbance peak. (**d**) Percentage of oxidized phenol for the two-vertical-loop microreactor as estimated by absorbance area.

**Figure 8:**
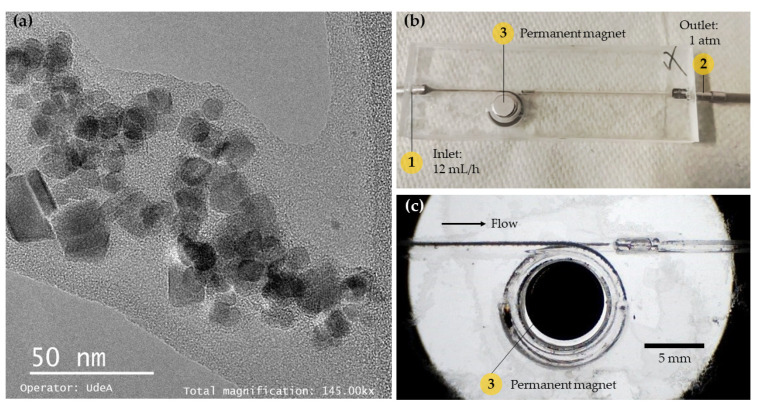
(**a**) Transmission electron microscopy micrograph of magnetite nanoparticles. (**b**) Picture of the one-loop microreactor during Eriochrome Black T dye treatment. (**c**) Picture of the one-loop microreactor during Eriochrome Black T dye treatment under optical microscope (10X magnification).

Potential for dye retention on the surface of the microreactor´s walls was estimated by pumping the dye solution into the system in the absence of magnetite. The average retention obtained was 24.3%, 52.1%, and 26.2% for the three dye concentrations evaluated (i.e., 5, 10, and 20 mg/L). Figure 7 shows the average removal of EBt for each device, condition, and concentration evaluated. Data are presented for the two types of analysis implemented in this work, i.e., absorbance peak and area under the absorbance spectrum. For almost all treatments, maximum removal/decolorization was achieved with the laccase-magnetite except for one-loop at 10 mg/L and two-horizontal loop at 20 mg/L, where bare magnetite nanoparticles were more effective in removing the dye. We attributed this superior performance to the adsorption of the dye molecules on the surface of the nanoparticles. The maximum removal of dye was 98.74% (4.9 mg/g dye/laccase-magnetite) for the two-vertical-loop microreactor. The average removals for laccase-magnetite systems based on absorbance (545 nm) (Figure 7a) were 91.08% (10.5 mg/g dye/laccase-magnetite), 74.38% (6.9 mg/g dye/laccase-magnetite) and 96.21% (11.1 mg/g dye/laccase-magnetite) for the one-loop, two-horizontal-loop, and two-vertical-loop microreactors, respectively. The dye removals for the same treatments but with quantification based on the area under the absorbance spectrum (Figure 7b) approached 85.62% (10.0 mg/g dye/laccase-magnetite), 62.44% (4.7 mg/g dye/laccase-magnetite), and 93.56% (10.7 mg/g dye/laccase-magnetite) for the three microreactors. Figure 8 shows a TEM micrograph of the magnetite, describing a size diameter ranging from 10 to 20 nm approximately, suggesting that the hydrodynamic diameter calculated via DLS is based on the agglomeration of nanoparticles in solution. In addition, Figure 8b,c show pictures of the operation of the one-loop microreactor during the Ebt removal process. Taken together, these results strongly suggest that the microreactor with the best performance in terms of the dye removal was the two-vertical-loop system. Additionally, in the case of the laccase-magnetite treatment, the overall (absorbance peak and area under the absorbance spectrum) removal efficiencies for this microreactor approached 98.05% (4.9 mg/g dye/laccase-magnetite), 93.87% (9.3 mg/g), and 92.74% (18.5 mg/g) for the low, medium, and high dye concentrations (5, 10, and 20 mg/L). The initial ratio between mass of laccase and dye for each experiment, corresponded to 2.6, 1.3 and 0.65 µg/mg, and the ratio between activity of laccase and dye mass was 0.00192, 0.00096 and 0.00048 U/mg, for the dye concentrations mentioned above.

Phenol removal was evaluated using the two-vertical-loop system (best performing microreactor from the three evaluated), at three initial phenol concentrations (5, 10 and 20 mg/L). Data (Figure 7c,d) are presented for the two types of analysis implemented in this work i.e., absorbance peak and area under the absorbance spectrum. The maximum phenol biotransformation was approximately 75% (3.75 mg/g phenol/laccase-magnetite) for the lowest phenol concentration, followed by approximately 50% (10 mg/g) at the highest concentration, and approximately 35% (3.5 mg/g) at the middle concentration. The initial ratio between mass of laccase and phenol for each experiment corresponded to 2.6, 1.3, and 0.65 µg/mg, and the ratio between activity of laccase and phenol mass was 0.00192, 0.00096, and 0.00048 U/mg, for the phenol concentrations mentioned above. Further research is needed to identify the potential intermediates that result from the transformation of the dye or phenol, their potential toxicity, and the potential need to include additional systems carrying other enzymes to complete the biodegradation process.

#### 3.3.2. Treatment of Real Wastewater

Phenol containing real wastewater (1 L) was treated through the two-vertical-loop microreactor in the presence of laccase-magnetite. Table 2 shows the measured water quality parameters before (U-RW) and after (T-RW) treatment. The removal of phenol reached 17.86% (700 mg/g phenol/Laccase-Magnetite), achieving a final concentration of 16.10 mg/L, which is close to but still greater than the Drinking Water Equivalent Level maximum (DWEL) in the U.S. i.e., of 11 mg/L [43] as imposed by the United States Environmental Protection Agency (EPA) regulations. Relatively low oxidation of phenol was expected due to the presence of very high concentrations of biodegradable organic matter (BOD) in real wastewater and its simultaneous oxidation by the laccase-magnetite system. It also needs to be considered that the initial ratio between mass of laccase and phenol for this experiment was only 0.00325 µg/mg, and the ratio between activity of laccase and phenol mass was 0.00000024 U/mg, representing 800-fold less than the ratio used for the dye and artificial wastewater experiments at the lower concentrations, or 200-fold reduction for the highest dye or artificial wastewater phenol concentration.

Recent studies have evaluated systems for treating large volumes of phenol-contaminated wastewater. These reports indicate that the most successful phenol remediation strategies for real wastewater rely on batch biodegradation processes [24]. For instance, Garg et al. reported the use of low-purity peroxidases extracts for the removal of phenol from textile industry wastewater in a batch process with an efficiency of 94.95 ± 0.82%. Additionally, the same process led to 91.49 ± 1.54% removal efficiency in leather industry wastewater [44]. Hayati et al. reported a removal ratio of 85.9% (TOC) and 92.1% (COD), with GZnTi nanocomposites [45]. However, studies that evaluate the transformation of phenol in continuous systems are rather scarce. Therefore, the observed reductions of phenol in our continuous system are promising and encourage us to continue exploring further improvements to the microreactors in continuous flow for future contributions. Moreover, to our knowledge, no other microreactor system has been identified in the literature capable of continuous operation for phenol-contaminated water treatment that treats large volumes of real wastewater and that also offers the possibility for in-line and real-time monitoring.

As there are amine groups present in the molecules used as linkers to complete the immobilization process, the nitrogen content becomes a parameter of interest. According to the results of two different techniques, i.e., NH_3_-nitrogen and Kjeldahl nitrogen, removal reached 16.67% and 20.16%, respectively, indicating that there was organic nitrogen oxidation due to laccase, but also that the nitrogen present in the linkers of the immobilization method were not released or lost during the treatment. This indicates that the functionalization of the laccase-magnetite was successful. Additionally, as the emission limit value (ELV) for nitrogen in wastewater by the regulation presented by the United States Environmental Protection Agency (EPA), is 10 mg/L and 15 mg/L [46], for NH_3_-nitrogen and Kjeldahl nitrogen, respectively; the treatment is likely helping in the nitrogen removal (final concentrations of 0.50 and 9.90 mg/L) as well. In the case of the biochemical oxygen demand (BOD), the removal was 10.39%, which allowed us to conclude that the treatment might this system can aid with the oxidation of BOD in real wastewater.

The initial phenol concentration of the real wastewater sample was 19.60 mg/L, which is close to the highest concentration evaluated in the case of biodegradation of dyes with model wastewaters. Additionally, the experiments with real wastewaters were also conducted in the presence of 5 mg of laccase-magnetite, but with a larger volume of wastewater than those carried out using dyes and phenol. Consequently, an equivalent number of cycles to treat the total volume of sample in each case can be estimated to be roughly 200.

In general, our results showed that our continuous flow treatment presented a maximum phenol oxidation of about 75% using nanocomposites, while other authors, as Hayati et al. [45], have reached phenol degradation, also using nanoparticles, between 90 and 100%. This higher phenol oxidation [45] was obtained in the presence of photocatalytic effects, and under non-continuous operation. The differences in the results might also be related to a longer reaction time and also the differences in the efficiency of a photocatalytic process, which requires also more external energy, compared to an enzymatic one. Abdollahi et al. [47] showed phenol degradation of 100% using nanoparticles and biocatalysis, a more related oxidation method to ours, however; it took between 4 and 12 h to reach those oxidation rates. Phenol oxidation was about 70 to 90% after 1 h, an oxidation rate similar to that obtained in our continuous process, which means that the efficiency might be highly related to the reaction time. Their results [47] also showed that their method lost 50% of the efficiency after 7 cycles, while our experiments with real wastewater, which represented roughly 200 cycles, reported lost 25% of the initial efficiency. Unfortunately, to our knowledge, there are no reports of microfluidic systems tested at such high number of cycles.

## 4. Conclusions

The removal of contaminants from industrial effluents remains a major challenge, thus, we aimed to evaluate laccase enzymes immobilized on magnetite nanoparticles (i.e., bionanocomposites) by controlling their interactions with real and synthetic wastewaters with the aid of low-cost microfluidic toroidal microreactors equipped with loops to accommodate permanent magnets that take advantage of the strong magnetic response of magnetite to control the residence time of laccase based bionanocomposites within the device without inducing detrimental perturbations. Multiphysics simulations indicated that the movement of the particles induced mixing patterns that can increase the interactions between the wastewater and the bionanocomposite. This is an improvement over large-scale torus reactors where mixing requires a mechanical stirring and significant energy consumption. The microreactor with the best overall performance (i.e., longest residence time and best interaction) was the two vertical loop system. This was corroborated experimentally by evaluating the biotransformation dyes in artificial wastewater, reaching efficiencies above 90%, and phenol removal from synthetic wastewater reaching efficiencies of about 80%. Real wastewater tests showed a decrease in phenol removal efficiency to about 18%, but using the same amount of bionanocomposites but with 200-fold the volume of the treated wastewater, representing the equivalent of 200 biodegradation operating cycles. Then, it would be worth exploring the idea of expanding the process for newer and more sustainable enzyme-based biodegradation routes enabled by microreactors [48]. Next steps should focus on exploring strategies for sustained and continuous operation, including recirculation and parallelization processing schemes. We also believe that it is worth exploring other microreactor geometries where a dynamic array of magnets can be tuned to maximize magnetic gradient and other geometry mixing approaches. Finally, based on the superior performance, we are exploring scaling-up routes and processing schemes and whether with such approaches key environmental indexes are positively impacted, while maintaining profitability.

## Figures and Tables

**Table 1:** Retention ratio analysis at the microreactor’s outlet after 30 s of simulation.

Remanent Flux Density ((mT))	Particle’s Loss Ratio (%)	Particle’s Retention Ratio (%)
0	93.67	6.33
50	91.17	8.83
100	85.00	15.00
200	56.00	44.00
300	17.33	82.67
350	3.17	96.83
500	0.00	100.00
1000	0.00	100.00

**Table 2:** Characterization of the large volume samples of real wastewater treated with the developed bionanocomposites.

Parameter	Sample			Removal Ratio (%)	Sorption Capacity (mg/g)
	U-RW	T-RW	Units		
Phenols	19.60	16.10	mg Phenol/L	17.86	700
Biochemical oxygen demand (BOD)	385.00	345.00	mg O_2_/L	10.39	8000
NH_3_ nitrogen	0.60	0.50	mg N/L	16.67	20
Kjeldahl nitrogen	12.40	9.90	mg N/L	20.16	500

## Data Availability

The data and contributions presented in the study are included in the article. Further inquiries can be directed to the corresponding author.
